# Comparison Effects of Propofol-Dexmedetomidine versus Propofol-Remifentanil for Endoscopic Ultrasonography: A Prospective Randomized Comparative Trial

**DOI:** 10.1155/2022/3305696

**Published:** 2022-11-07

**Authors:** Liang Zhao, Yonglai Zhang, Shoucai Xu, Xiuqin Wang

**Affiliations:** ^1^Department of Anesthesiology, The 960th Hospital of Joint Logistics Support Force of Chinese People's Liberation Army, Jinan, Shandong 250031, China; ^2^Department of Anesthesiology, Shandong Cancer Hospital, Shandong Cancer Hospital and Institute Affiliated to Shandong First Medical University (Shandong Academy of Medical Science), Jinan, Shandong 250117, China

## Abstract

**Objective:**

To compare the effects of propofol-dexmedetomidine versus propofol-remifentanil for endoscopic ultrasonography (EUS). *Design, Setting, and Participants.* A single-center, randomized trial from August 20, 2020 to August 20, 2021, in patients undergoing EUS. *Interventions.* Propofol-dexmedetomidine (PD) versus propofol-remifentanil (PR). *Outcome Measures.* The primary outcome was the endoscopist satisfaction level. The secondary outcomes included patient satisfaction, the incidence of adverse events, induction time, and time to achieve postanesthesia discharge score (PADS) ≥9.

**Methods:**

Total of 200 patients were enrolled and randomized into PD and PR groups. A bolus dose of 0.5 *μ*g/kg dexmedetomidine was injected intravenously for 5 min. Subsequently, a continuous infusion of 0.5 *μ*g/kg/h for the PD group. Remifentanil was continuously infused at 1.5 *μ*g/kg/h for the PR group. A bolus dose of 1 mg/kg propofol was administered to both groups and then continuously infused.

**Results:**

The endoscopist satisfaction level was higher in the PR group than in the PD group (*P* = 0.009). Patient satisfaction was not significantly different between the groups (*P* = 0.738). No patients required mask ventilation or tracheal intubation in both groups. All patients were relatively hemodynamically stable. The incidence of body movements during the procedure in the PD group was higher than in the PR group (*P* = 0.035). The induction time and time taken to achieve PADS ≥9 in the PD group were longer than in the PR group (*P* < 0.05).

**Conclusions:**

PR sedation can increase the satisfaction level of the endoscopist by providing faster induction time and lower body movement and that of the patient by achieving faster PADS than PD sedation. Trial registration number: http://www.chictr.org.cn (ChiCTR2000034987).

## 1. Introduction

Gastrointestinal (GI) endoscopy provides a diagnostic and therapeutic opportunity for the management of digestive tract diseases [1–3]. Endoscopic ultrasound (EUS) is an advanced endoscopic procedure, which can obtain an endoluminal image and provide definite staging of gastroenterological cancer [4, 5]. EUS procedures use endoscopes with larger diameters (13.8–14.6 mm) than those used in conventional esophagogastroduodenoscopy (9.9–10.2 mm) [6]. However, this procedure is associated with greater and longer discomfort for patients than general GI endoscopy. EUS may require higher doses of sedatives and analgesics to allow patient comfort and compliance, thus increasing the risk of sedation along with prolonged recover [7, 8].. Propofol is a commonly administered agent for procedural sedation due to its rapid onset and offset that allows for a timely recovery [9]. However, significant and possibly poorly tolerable adverse effects such as hypotension, tachycardia, and apnea can occur when large doses are required to complete a procedure [10].

Enhanced recovery protocols employ one or more approaches to improve the clinical outcomes of a procedure. For EUS, it might involve combining several agents with different mechanisms of action to achieve the desired level of sedation and minimize individual adverse side effects [11, 12]. Dexmedetomidine is a selective alpha-2-adrenoceptor agonist with sedative and analgesic effects that has been successfully used during GI endoscopy [13]. Remifentanil is a modestly sedative ultrashort-acting opioid analgesic with unique pharmacokinetic properties [14]. There have been many studies investigating propofol in combination with other agents for GI endoscopy, but to our knowledge, only a few of them have been examined for EUS. We hypothesized that the efficacy and safety between PD and PR group were comparable. As a result, this trial was conducted to compare the efficacy and safety of propofol-dexmedetomidine (PD) with propofol-remifentanil (PR) during EUS.

## 2. Materials and Methods

### 2.1. Study Design and Setting

**Figure 1:**
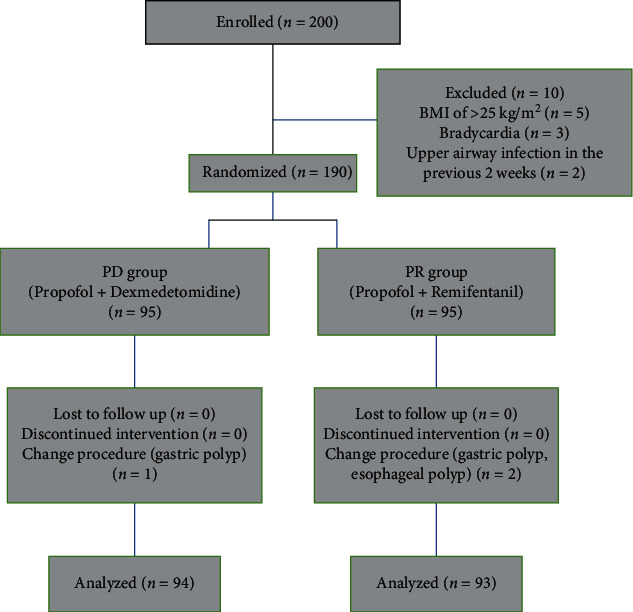
Consolidated standards of reporting trials flow diagram.

This randomized blinded comparative trial was approved by the Medical Ethics Committee of the Shandong Cancer Hospital and Institute affiliated with Shandong First Medical University (2020008001, Jinan, Shandong, China) and was registered at http://www.chictr.org.cn (ChiCTR2000034987). Written informed consent was obtained from all patients before the procedure. Patients aged 18–70 years with the American Society of Anesthesiology score of I–III and scheduled for EUS were enrolled from August 20, 2020 to August 20, 2021. Patients with a body mass index (BMI) of >25 kg/m^2^ or<18 kg/m^2^, serious comorbidities (i.e., heart failure, respiratory failure, hepatic, or renal failure), bradycardia with cardiac conduction or rhythm abnormalities, history of bronchial asthma or chronic obstructive pulmonary disease, upper airway infection in the previous 2 weeks, sleep apnea, known allergy to drugs used in the trial, history of drug abuse, and pregnant women were excluded from the study. A computer-generated randomization table (provided by http://www.random.org) was used to divide patients into PD and PR groups in a 1 : 1 ratio by an independent research staff member (Figure 1). Allocations were concealed in sequentially numbered and sealed opaque envelopes, which were opened the day before surgery after patients had consented to the trial.

An independent nurse anesthetist dispensed premade, numbered syringes containing either dexmedetomidine (Hengrui Pharmaceutical Co., Ltd., Jiangsu, China, 200 *μ*g) or remifentanil (Humanwell Pharmaceutical Co., Ltd., Yichang, China, 1 mg) dilution to 50 mL with 0.9% saline, according to the table. This procedure ensured that the patient, staff involved in data collection, endoscopists, and clinical anesthetists were blinded to the actual content of each syringe and unaware of the group assignment. The infusion pump was back to the patient and endoscopist. So they could not know how the research staff adjusted the drugs during the procedure. Only the research staff and nurse anesthetist knew to which group each patient was assigned. All EUS procedures were performed by an expert GI endoscopist who was blinded to the groups.

### 2.2. Anesthesia

Peripheral intravenous access was secured using a 20-gauge intravenous catheter on the dorsum of the right hand before the patients entered the operating room. All patients were placed in the left lateral position before sedation. Sedation was assessed using the Modified Observer's Assessment of Alertness/Sedation Scale [15] (MOAA/S) and was targeted to a score of 2–3 and bispectral index (BIS) value of approximately 60 for moderate sedation during the procedure. Electrocardiogram (ECG), pulse oximetry (SpO_2_), and noninvasive blood pressure were continuously monitored and recorded at 5 min intervals. The baseline vital signs were recorded before the procedure. Oxygen was administered via a nasal cannula (3 L/min). Normal saline was infused intravenously at a rate of 3–5 mL/kg/h during the procedure. A bolus dose of 0.5 *μ*g/kg dexmedetomidine was injected intravenously for 5 min and then continuously infused at a dose of 0.5 *μ*g/kg/h for the PD group. Remifentanil was continuously infused at a dose of 1.5 *μ*g/kg/h for the PR group. After dexmedetomidine was injected for 5 min in the PD group (remifentanil in PR group, accordingly), a bolus dose of 1 mg/kg propofol (Diprivan; Astra Zeneca, Australia) was injected in both groups. The same nurse anesthetist infused the drugs. When the MOAA/S scale score was 2 or 3, and the BIS reached 60, the endoscope was then inserted. Propofol was followed by a continuous infusion of 4 mg/kg/h. A bolus of 10 mg propofol was administered when the patient experienced discomfort, or the endoscopist noted body movements or reflex coughing, and its infusion rate was increased by 1 mg/kg/h. A BIS value of approximately 60 was chosen to target the levels of sedation that could be considered at both moderate sedation and general anesthesia [16]. Desaturation (SpO_2_<90%) was managed by chin lift or jaw thrust maneuver. If these methods failed or if SpO_2_ dropped to <85%, the procedure was terminated, and mask ventilation was used. Cases requiring placement of an advanced airway were classified as major adverse events. Hemodynamic monitors maintained the variation range of mean arterial pressure (MAP) and heart rate (HR) within 20% of baseline values. Equipment for full resuscitation was available at all times within the endoscopy unit. Naloxone was used as a reversal agent when severe adverse effects resulted from the use of remifentanil. At the end of the procedure, patients were assessed using the MOAA/S scale and transported to the postanesthetic care unit (PACU). All patients were observed in the PACU, and the modified postanesthetic discharge scoring system [17] was used to assess the patient's general status until a postanesthesia discharge score (PADS) ≥9 was achieved.

### 2.3. Outcome Measurements

The primary outcome was to compare the level of satisfaction of the endoscopist, which was assessed at the end of the procedure on a verbally administered numerical rating scale of 0 to 10, where 0–3 indicated a low level of satisfaction; 4–6 indicates medium satisfaction; 7–10 indicates high satisfaction. Secondary outcomes included the incidence of cardiopulmonary adverse events, patient satisfaction level for the procedures, the incidence of body movement, reflex coughing, postoperative nausea and vomiting, and the induction time (marked from the time of administration of injecting the anesthetic until a MOAA/S scale score 2–3). Cardiopulmonary adverse events were defined as an SpO_2_ of <90%, a MAP of <65 mmHg or>100 mmHg, or HR <50 beats/min or>120 beats/min. Adverse respiratory events were managed with positional maneuvers (chin lift or jaw thrust), mask ventilation, or tracheal intubation. Significant bradycardia or tachycardia and hypotension or hypertension were treated with glycopyrrolate 0.2 mg or esmolol 0.5 mg/kg and ephedrine 5–10 mg or nitroglycerin via intravenous boluses, respectively. Intraoperative medications and total dosages of propofol, dexmedetomidine, and remifentanil were recorded. The MOAA/S scale score and the BIS value were recorded as follows: immediately before the procedure (baseline, T0); 1 min after the induction of sedation (1 min after a 5 min loading of dexmedetomidine in the PD group and remifentanil in PR group, respectively (T1); as the endoscope was passed into the esophagus (T2); as the ultrasound endoscope infiltrated the tumor region (T3); before ultrasound endoscope removal (T4); at the end of the procedure (T5). Procedures performed, duration of the procedure (the time interval from the insertion of the endoscope to the end of the procedure), PADS at the end of the procedure, awakening time (from endoscope removal to patient reaching a MOAA/S scale score of 5), and the time from end-of-procedure to achieve PADS of ≥9 were also recorded. Before discharge, the visual analog scale pain score (VAS) was assessed using an 11-point scale (0–3, mild pain; 4–6, moderate pain; 7–10, severe pain). The patient satisfaction level for the procedure was assessed (same manner as the endoscopist), and all patients were asked whether they experienced any recall regarding the procedure.

### 2.4. Statistical Analysis

The sample size of the study was calculated using the *χ*^2^ test based on the results of our pilot study. The number of patients according to the satisfaction level was as follows: one patient with a low satisfaction score (0–3), one with medium satisfaction (4–6), and eight with high satisfaction (7–10) in the PR group; one patient with a low satisfaction score (0–3), three with medium satisfaction (4–6), and six with high satisfaction (7–10) in PD group. Based on these results, the effect size was estimated as 0.25. In addition, the degree of freedom was 2 because values of the primary outcome were classified into a 2×3 contingency table. Therefore, a sample size of 155 was required to achieve an 80% power to detect an effect size of 0.25 using the *χ*^2^ test with 2 degrees of freedom and a significance level of 0.05. We recruited 200 patients considering a 20% dropout rate.

Data were analyzed using SPSS version 22.0 (IBM Corporation, Armonk, NY, USA) for Windows (Microsoft Corporation, Redmond, WA, USA). All data were assessed for normal distribution using the Kolmogorov–Smirnov test and histograms. Data are presented as means ± standard deviation (SD) for normally distributed continuous variables or frequencies (percentages) for categorical variables. Continuous data with normal distribution were compared using the independent sample *t*-test. Categorical data were compared with the *χ*^2^ test or Fisher's exact test. Correlation analyses were performed to identify the strength of the relationship between high satisfaction of the endoscopist and body movement and induction time. A *P* value of < 0.05 was considered statistically significant.

## 3. Results

### 3.1. Study Population and Baseline Characteristics

A total of 200 adult patients scheduled for elective EUS under anesthesia were enrolled. Ten patients were excluded because of the following reasons: BMI of >25 kg/m^2^ (*n* = five), bradycardia (*n* = three), and upper airway infection in the previous 2 weeks (*n* = 2). Finally, 190 patients (95 in each group) were examined. However, one patient in the PD group and two patients in the PR group were eventually excluded due to a change in the end procedure. Thus, data on 94 patients in the PD group and 93 patients in the PR group were considered in the results of this study (Figure 1). The procedures included EUS alone and EUS+ fine needle aspiration cytology (FNAC). There were 61 (64.9%) and 33 (35.1%) patients in the PD group and 58 (62.4%) and 35 (37.6%) patients in the PR group undergoing EUS alone or EUS+FNAC, respectively (*P* = 0.762). There were 20 and 26 patients in the PR and PD groups who required additional propofol dosage, respectively (*P* = 0.396). The procedural time was not significantly different between the two groups. The baseline characteristics of the patients were comparable between the two groups (Table 1).

### 3.2. Safety Profiles and Medications Used

**Figure 2:**
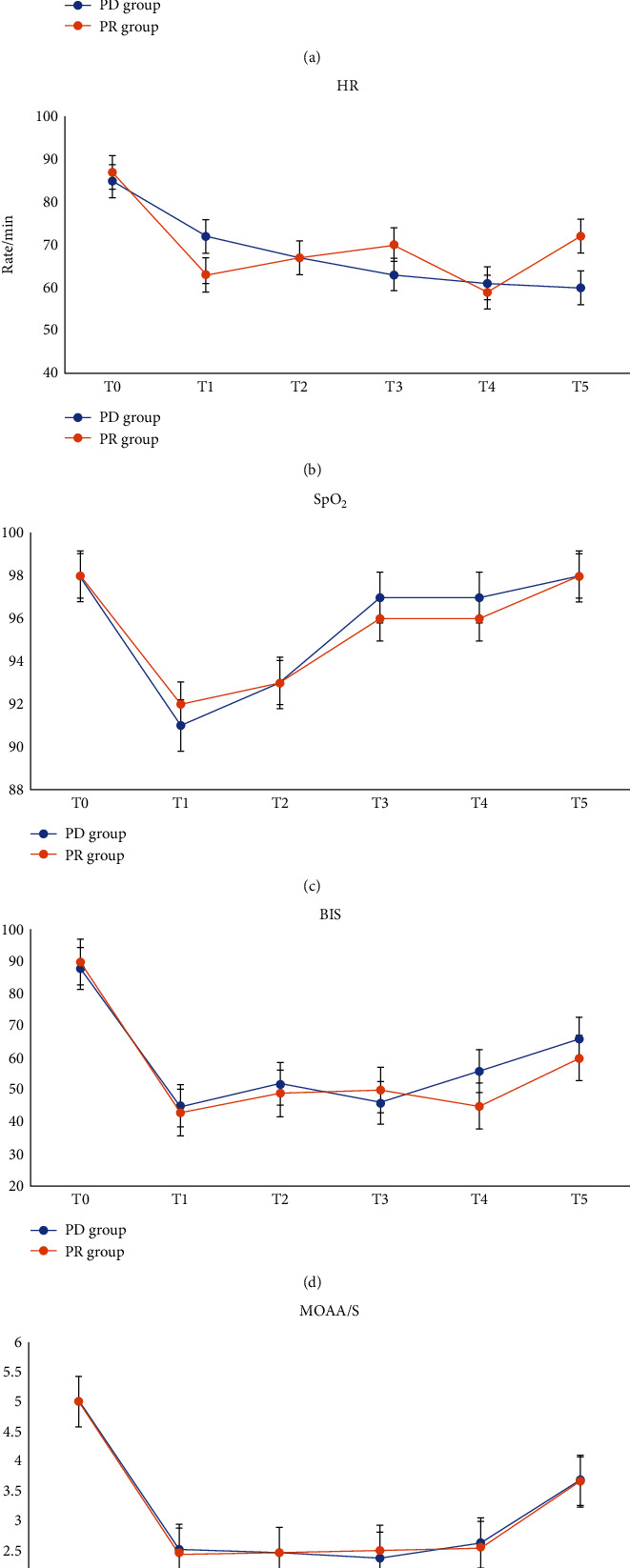
Changes in the intraoperative MAP, HR, SpO_2_, and BIS. T0: just before the procedure; T1: 1 min after induction of sedation (1 min after a 5 min loading of dexmedetomidine in the PD group and remifentanil in PR group, respectively); T2: as the endoscope was passed into the esophagus; T3: as the ultrasound endoscope got to the tumor region; T4: before ultrasound endoscope removal; T5: and at the end of the procedure. PD group: propofol-dexmedetomidine group; PR group: propofol-remifentanil group; MAP: mean arterial blood pressure; HR: heart rate; SpO_2_: pulse oxygen saturation; BIS: bispectral index.

Three patients in each group required chin lift or jaw thrust maneuvers because of desaturation (SpO_2_<90%). No patients required mask ventilation or tracheal intubation in both groups. All patients were relatively hemodynamically stable. Ephedrine was administered to one patient from each group. Bradycardia was noted in two patients in the PD group and was treated with glycopyrrolate, and one patient developed hypotension, which resolved after ephedrine administration. Similarly, one patient in the PR group experienced hypotension associated with bradycardia and was treated with ephedrine. There were 20 and 26 patients in PD and PR groups who needed additional propofol dosage, respectively (*P* = 0.396). The additional and total dosages of propofol were not significantly different between the two groups (Table 2). Intraoperative monitoring data, including MAP, HR, SpO_2_, respiratory rate, BIS, and MOAA/S score, were comparable between the two groups (Figure 2).

### 3.3. Procedure-Related Findings, Side Effects, and Score

Induction time in the PD group was longer than that in the PR group (7.20 ± 0.63 vs 6.36 ± 0.52 min, respectively) (*P* = 0.012). The time taken to achieve a PADS ≥9 was 16.56 ± 3.91 min in the PD group, which was longer than that in the PR group at 15.39 ± 3.8 min (*P* = 0.035). The end-of-procedure PADS and awakening time were not significantly different between the two groups (Table 3).

The incidence of body movement was higher in the PD group than in the PR group (eight [8.51%] vs. one [1.08%] patient; *P* = 0.042). No patients experienced reflex coughing or nausea and vomiting in both groups. The VAS score was also comparable between the two groups (Table 3).

### 3.4. Level of Satisfaction

The level of satisfaction of the endoscopist was higher in the PR group than in the PD group (*P* = 0.009), whereas patient satisfaction was not significantly different between the two groups (*P* = 0.738) (Table 4). Correlation analyses were performed to identify the strength of the relationship between high satisfaction of the endoscopist and body movement and induction time. Both body movement and induction time had a negative correlation with the level of satisfaction of the endoscopist (*r* = −0.155, *P* = 0.034 and *r* = −0.57, and *P* < 0.001, respectively) (Table 5).

## 4. Discussion

This study was the first prospective randomized blinded comparative trial to compare the effects of PD versus PR titrated to provide moderate sedation for patients who underwent EUS. Although the efficacy and safety between PD and PR were comparable, our study noted that PR could be more favoring for endoscopists during EUS, and it was associated with faster induction and discharge time and lower incidence of body movements than PD.

Level of satisfaction is important for both endoscopists and patients in clinical practice. In our study, we found that patients in both groups were satisfied with the procedures. In contrast, endoscopists preferred the PR regimen. Correlation analyses revealed that both the body movement and induction time had a negative correlation with the level of satisfaction of the endoscopist. The reason remains unclear but may be related to the sedation characteristic of dexmedetomidine. In our study, we used 0.5 *μ*g/kg as a loading dose for dexmedetomidine. Recommended loading dose for dexmedetomidine is different depending on the age of patients. And we thought that 1 *μ*g/kg loading dose for 10 min may not suitable in our study because some procedure would be finished in 15-20 min.

The efficacy and safety of PD groups versus PR group were comparable in the study. We observed that if adequate analgesia was provided, EUS could be safely performed under MOAA/S levels of 2–3. Propofol is recommended for sedation in the EUS procedures [18]. However, minimizing propofol-associated adverse events due to high doses by combining propofol with other sedatives may be an optimal method [19]. Dexmedetomidine is recognized as one of the most preferred drug options but one of its adverse effects is bradycardia. Only two patients had bradycardia, and one was treated with glycopyrrolate and another patient developed hypotension, which resolved after ephedrine administration. Hypoxemia is one of the major respiratory complications of propofol-based sedation. We defined respiratory adverse events as an SpO_2_ of <90% requiring the use of chin lift or jaw thrust maneuvers to improve desaturation. Assisted ventilation was used, such as mask ventilation or tracheal intubation, if desaturation persisted. In this study, three patients in both groups experienced desaturation requiring chin lift maneuvers. Mask ventilation or tracheal intubation was unnecessary in both groups.

While some studies reported a lower HR in patients receiving dexmedetomidine, the values were generally above 50 beats/min and, as such, typically did not require any intervention to counteract bradycardia [20–22]. Recent studies [23, 24] suggested that maintaining a MAP of 65 mmHg was equivalent to the classic “20% rule” of maintaining the blood pressure within 20% of the preoperative values. We used continuous infusion remifentanil (1.5 *μ*g/kg/h) and propofol (4 mg/kg/h), which was close to the target-controlled infusion concentration, and our results were consistent with LaPiere et al.'s [25] findings. Furthermore, Kim et al. [26] found that the efficacy and safety of PR and dexmedetomidine-remifentanil during endoscopic submucosal dissection were comparable. Most studies revealed that dexmedetomidine provides sedation and analgesia without impairing the airway patency [21, 27]. Lodenius et al. [28] suggested that dexmedetomidine sedation might cause upper airway collapse. The difference in the findings may be due to dissimilarities in the population, the dosage of dexmedetomidine, and study objectives. Edokpolo et al. [29] demonstrated that combined low-dose dexmedetomidine with propofol delayed the discharge readiness for colonoscopy. Our findings were consistent with the reports of Chen et al. [30] and Ryu et al. [31], wherein PD was associated with a longer recovery time.

Although it is important to minimize the cost, the cost-benefit analysis between the two groups were not discussed here because of the international cost variations of medications.

Our study has several limitations. First, it was performed by one endoscopist at a single center, and therefore, these results could not be generalized. Multiple center-clinical trials will be required in the future. The way of inserting the endoscope may have affected the need to deepen anesthesia and we chose one endoscopist. Second, we did not assign a propofol-only group or a dexmedetomidine-remifentanil group. Only propofol administration for sedation in the GI endoscopy advanced procedure may be unfavorable. Currently, there is limited information on dexmedetomidine combined with remifentanil for advanced endoscopy procedure sedation. Thus, further direct interventional studies are necessary. Finally, our study did not measure the partial pressure of carbon dioxide using the arterial blood gas analysis or nasal oxygen cannula; therefore, we could not exclude the possibility of hypercapnia. Instead, we used the BIS value and MOAA/S scale to maintain a constant level of moderate sedation. Patients in both groups had a faster recovery from sedation, and the study population was limited among the healthier population due to stringent inclusion criteria in terms of comorbidities, thus limiting the possibilities of hypercapnia.

In conclusion, the combination of propofol with dexmedetomidine or remifentanil for EUS sedation is safe and efficacious. No statistically significant difference in the procedure time, awakening time, and propofol consumption were observed between the two groups. The PR regimen achieved a PADS of 9 faster than the PD regimen and was more favored by endoscopists due to the faster induction time and lower body movement.

## Figures and Tables

**Table 1:** Basic characteristics and demographics of procedures.

Characteristic	PD group (*n* = 94)	PR group (*n* = 93)	*P* value
Age, mean ± SD, *y*	57.53 ± 9.58	57.88 ± 8.64	0.794
Male/female	49/45	50/43	0.884
BMI, mean ± SD, kg/m^2^	20.65 ± 2.13	21.12 ± 2.22	0.587
ASA classification, *n* (%)			0.881
I	23 (24.5%)	20 (21.5%)	
II	65 (69.2%)	66 (71.0%)	
III	6 (6.3%)	7 (7.5%)	
Baseline MAP, mean ± SD, mmHg	87.06 ± 12.73	85.56 ± 12.56	0.418
Baseline HR, mean ± SD, beats/min	71.57 ± 9.96	71.70 ± 10.20	0.930
Baseline SpO_2_, mean ± SD	96.53 ± 1.12	96.40 ± 1.17	0.439
Type of procedure, *n* (%)			0.762
EUS alone	61 (64.9%)	58 (62.4%)	
EUS+FNAC	33 (35.1%)	35 (37.6%)	
Procedure time, min	25.67 ± 5.58	27.29 ± 6.23	0.063

Values are presented as means ± standard deviation or frequencies (percentages). PD group: propofol-dexmedetomidine group; PR group: propofol-remifentanil group; ASA: American Society of Anesthesiologists; BMI: body mass index; MAP: mean arterial blood pressure; HR: heart rate; SpO_2_: pulse oxygen saturation; EUS: endoscopic ultrasonography; EUS+FNAC: EUS guided fine needle aspiration cytology (FNAC).

**Table 2:** Respiratory and hemodynamic adverse events and medication.

	PD (*n* = 94)	PR (*n* = 93)	*P* value
Respiratory adverse events			
Chin lift/jaw thrust	3 (3.2%)	3 (3.2%)	1.000
Mask ventilation	0	0	
Tracheal intubation	0	0	
Hemodynamic adverse events			
Hypotension	8 (8.5%)	5 (5.4%)	0.567
Bradycardia	2 (2.1%)	1 (1.1%)	1.000
Usage of vasoactive drugs	1 (1.1%)	1 (1.1%)	1.000
Usage of glycopyrrolate	1 (2.1%)	0	1.000
Dosage of dexmedetomidine, *μ*g	50.31 ± 7.83		
Dosage of remifentanil, *μ*g		54.95 ± 13.90	
Additional propofol			
Patients, *n* (%)	26 (27.7%)	20 (21.5%)	0.396
Dosage, mg	16.81 ± 10.23	13.12 ± 6.06	0.183
Total dosage of propofol, mg	233.44 ± 44.79	244.65 ± 49.01	0.104

Values are presented as frequencies (percentages). PD group: propofol-dexmedetomidine group; PR group: propofol-remifentanil group.

**Table 3:** Procedure-related times, side effects, and score.

	PD (*n* = 94)	PR (*n* = 93)	*P* value
Induction time, min	7.20 ± 0.63	6.36 ± 0.52	<0.001
End-of-procedure PADS	5.76 ± 0.84	5.76 ± 0.81	0.696
Awakening time, min	3.56 ± 0.76	3.53 ± 0.84	0.753
Time to achieve PADS 9, min	16.56 ± 3.91	15.39 ± 3.8	0.035
Body movement (%)	8 (8.5%)	1 (1.1%)	0.035
Reflex coughing (%)	0	0	1.000
Nausea and vomiting (%)	0	0	1.000
VAS score	0.12 ± 0.23	0.18 ± 0.29	0.139

Values are presented as means ± standard deviation or frequencies (percentages). PD group: propofol-dexmedetomidine group; PR group: propofol-remifentanil group; PADS: postanesthesia discharge score; VAS: visual analog scale.

**Table 4:** Satisfaction scores of patients and endoscopist.

	PD (*n* = 94)	PR (*n* = 93)	*P* value
Satisfaction of patients			0.738
High	64 (68.08%)	68 (73.12%)	
Medium	28 (29.79%)	23 (24.73%)	
Low	2 (2.13%)	2 (2.15%)	
Satisfaction of endoscopist			0.009
High	43 (45.75%)	42 (45.16%)	
Medium	40 (42.55)	50 (53.76%)	
Low	11 (11.70%)	1 (1.08%)	

Values are presented as frequencies (percentages). PD group: propofol-dexmedetomidine group; PR group: propofol-remifentanil group.

**Table 5:** Associate factor for affecting satisfaction score of endoscopist.

Variable	*n*	High-level satisfaction scores *n* (%)	*r*	95% CI	*P* value
Body movement			-0.155	-(0.292–0.011)	0.034
Yes	9	1 (11.1)			
No	178	84 (47.2)			
Induction time			-0.57	-(0.659–0.463)	<0.001
≥7 min	82	15 (18.3)			
<7 min	105	70 (66.7)			

Values are presented as frequencies (percentages). PD group: propofol-dexmedetomidine group; PR group: propofol-remifentanil group; CI, confidence interval.

## Data Availability

Data are available on request through the corresponding author Xiuqin Wang. The email is wangxiuqin@sdfmu.edu.cn, and the website is http://mail.sdfmu.edu.cn/.
